# 8-Oxoguanine Forms Quartets with a Large Central Cavity

**DOI:** 10.1021/acs.biochem.2c00478

**Published:** 2022-10-19

**Authors:** Simon Aleksič, Peter Podbevšek, Janez Plavec

**Affiliations:** † Slovenian NMR Centre, National Institute of Chemistry, Hajdrihova 19, 1000 Ljubljana, Slovenia; ‡ Faculty of Chemistry and Chemical Technology, University of Ljubljana, Večna pot 113, 1000 Ljubljana, Slovenia; § EN-FIST Centre of Excellence, Trg OF 13, 1000 Ljubljana, Slovenia

## Abstract

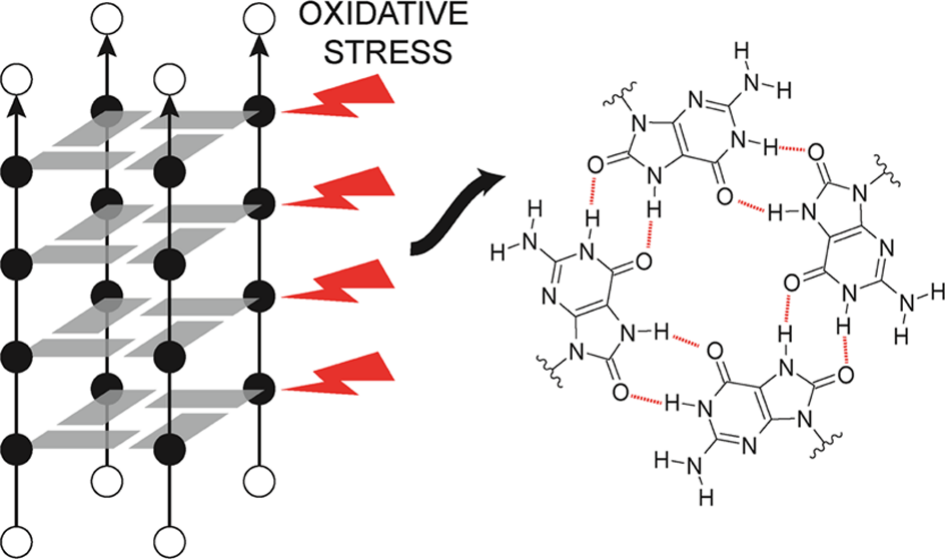

Oxidation of a guanine nucleotide in DNA yields an 8-oxoguanine nucleotide (^oxo^G) and is a mutagenic event in the genome. Due to different arrangements of hydrogen-bond donors and acceptors, ^oxo^G can affect the secondary structure of nucleic acids. We have investigated base pairing preferences of ^oxo^G in the core of a tetrahelical G-quadruplex structure, adopted by analogues of d(TG_4_T). Using spectroscopic methods, we have shown that G-quartets can be fully substituted with ^oxo^G nucleobases to form an ^oxo^G-quartet with a revamped hydrogen-bonding scheme. While an ^oxo^G-quartet can be incorporated into the G-quadruplex core without distorting the phosphodiester backbone, larger dimensions of the central cavity change the cation localization and exchange properties.

## Introduction

Guanine-rich repeats are abundant in telomeres and promoter regions of genomes, where they can form non-B DNA structures.^1^ Four guanines can form a G-quartet, stabilized by hydrogen bonds in the Hoogsteen geometry. G-quartets stack with each other to form the core of a G-quadruplex. In tetrameric G-quadruplexes, four oligonucleotides adopt parallel or antiparallel directionalities, and guanine nucleotides adopt either *anti* or *syn* conformations.^2^

Guanine has the lowest redox potential of the four nucleobases found *in vivo* and is thus most likely to get oxidized. G-rich repeats are even more susceptible to oxidation.^3^ Reactive oxygen species (ROS), which are a byproduct of cellular respiration, can induce oxidation of the guanine moiety to yield 8-oxoguanine nucleotides (^oxo^G), among other oxidation products.^4,5^ It is believed that G-rich regions act as oxidation sinks, thus serving as DNA damage reservoirs.^6,7^^oxo^G can pair with both adenine and cytosine in a DNA duplex and thus cause G to T transverse mutations.^8^ When paired with adenine, ^oxo^G adopts the *syn* conformation in B-DNA duplexes as shown by NMR,^9^ X-ray diffraction,^10^ and molecular dynamics.^11^ Similarly, ^oxo^G adopts the *syn* conformation in G-quadruplexes originating from human telomeres, leading to structure reorganization.^12,13^^oxo^G has recently been found to accumulate in enhancer regions of the human genome, with the oxidized enhancers also being enriched with G-quadruplex structures.^14^ However, we are far from understanding how the incorporation of ^oxo^G into G-rich oligonucleotides affects the formation of G-quadruplexes and their thermal stability.^12,13,15−17^ Since G-quadruplex forming sequences are found in regulatory regions of the genome and in telomeres, a change in the structure and/or stability of G-quadruplexes can affect cellular processes such as replication, transcription, and telomere maintenance.^18−20^

Our recent studies revealed that the introduction of a single ^oxo^G into G-rich constructs originating from human telomeres and oncogene promoter regions does not necessarily prevent G-quadruplex formation.^13,21^ Three scenarios were found to accompany ^oxo^G incorporation. Certain positions were found to tolerate ^oxo^G substitutions while retaining the original G-quadruplex topology. Alternatively, substitutions with ^oxo^G induced changes in strand directionality and/or rearrangements in G-quadruplex loops.^13^ Both scenarios resulted in suboptimal hydrogen bonding of ^oxo^G and a substantial reduction of thermal stability.^21^ However, in the third scenario, ^oxo^G was displaced from the G-quadruplex core and formed well-stacked (wobble) base pairs with loop nucleotides, which enhanced the thermal stability of the G-quadruplex structure.

This work focuses on the evaluation of structural effects and changes in thermal stability caused by the incorporation of ^oxo^G in a simple G-quadruplex model system without interference of loop interactions. A model system was chosen to discern the effects of ^oxo^G incorporation that originate from specific stacking and/or hydrogen bonding between ^oxo^G and adjacent G nucleotides. For this purpose, we utilized the d(TG_4_T) oligonucleotide, which forms a parallel tetrameric G-quadruplex with four G-quartet planes and thymine overhangs on 5′ and 3′ ends.^22,23^ It was previously shown that G to ^oxo^G substitutions within d(TG_4_T) generally decrease the thermal stability of G-quadruplex structures in a Na^+^ cation solution; however, when substituting the third guanine position in d(TG_4_T), a slight increase in thermal stability of the G-quadruplex structure was observed.^15^ Mixed ^oxo^G-G quartets could be achieved either by reversal of directionality of two strands resulting in antiparallel G-quadruplexes or by slipping of two strands, while retaining the parallel topology. Our initial hypothesis was that mixed quartets, including both G and ^oxo^G nucleotides, would induce less perturbations in the structure and be preferred over quartets composed exclusively of ^oxo^G. The affinity of ^oxo^G for the *syn* glycosidic conformation could favor strand reversal and influence stacking interactions within individual strands.

## Experimental Details

### Oligonucleotide Synthesis and Sample Preparation

Oligonucleotides were synthesized using a DNA/RNA H-8 Synthesizer (K&A Laborgeräte) operating on the phosphoramidite chemistry principle and using nucleotide phosphoramidites obtained from Glen Research. All oligonucleotides were synthesized with DMT protecting group. Deprotection and deblocking were achieved with ammonium hydroxide and methylamine in a 1:1 (v/v) ratio for 30 min at room temperature and 30 min at 65 °C. Samples were purified using GlenPak cartridges and desalted on FPLC with a HiPrep 26/10 Desalting column (GE Healthcare). Samples were dried using a vacuum centrifuge and dissolved in 1 mL of deionized water and dialyzed against 110 mM NaCl or KCl overnight. After dialysis, ^2^H_2_O was added to a final 10% concentration. Solutions were buffered at pH 7 using either NaPi for Na^+^ solution or KPi for K^+^ solution. The oligonucleotide solutions were heated to 95 °C for 5 min and left to cool at room temperature. For mixed Na^+^/K^+^ cation samples, 100 mM KCl samples were diluted 2-fold and sodium chloride solution and ^2^H_2_O were added to a final concentration of 50 mM NaCl, 50 mM KCl, 5 mM KPi pH 7, and 90%/10% ^1^H_2_O/^2^H_2_O. Concentration of monomers in solution was determined by UV spectrophotometry using a Varian Cary 100 Bio UV/VIS spectrophotometer at a wavelength of 260 nm, using the extinction coefficient ε = 57,800 M^–1^ cm^–1^ for all oligonucleotides. The extinction coefficient was determined by the nearest neighbor method. The concentration of oligonucleotides per strand was between 0.5 and 1.0 mM.

### CD Spectroscopy

NMR samples of ODN2-5 in a mixture of 50 mM NaCl and 50 mM KCl were kept at NMR concentration and their CD spectra were recorded on a Chirascan CD spectrometer (Applied Photophysics) at 25 °C. Spectra were recorded at wavelengths from 220 to 320 nm with a 0.1 mm cell length in 10 parallels.

### UV Melting

We diluted 30 μL of Na^+^/K^+^ NMR sample in 970 μL of buffer solution (50 mM NaCl, 50 mM KCl, 5 mM potassium phosphate, pH 7). We recorded absorbance at 260 nm wavelength from 15 to 95 °C, with a temperature gradient of 0.5 °C/min and sampling at every 0.1 °C. We determined the first-order derivatives using Origin 2018.

### NMR Spectroscopy

NMR data were collected on Bruker Avance NEO 600 and 800 MHz NMR spectrometers. One-dimensional (1D) and two-dimensional (2D) NMR spectra were acquired using excitation sculpting to achieve solvent suppression.

### Molecular Dynamics

Molecular dynamics calculations were performed with the AMBER 20 software using the ff99bsc0 force field and ε/ζOL1 and χOL4 modifications. Force field parameters for ^oxo^G nucleotides were obtained from the R.E.D. Server. Calculations were started from initial linear structures obtained with the LEAP module of AMBER 20. A total of 100 structures were obtained in 1 ns restrained simulated annealing simulations using the Born implicit solvent model with random starting velocities. Restraints included hydrogen-bond distances in G- and ^oxo^G-quartets and χ torsion angles. Force constants were 20 kcal·mol^–1^·Å^–2^ for hydrogen bonds and 200 kcal·mol^–1^·rad^–2^ for torsion angles. In the first 200 ps, the temperature was held at 1000 K. The temperature was decreased to 300 K in the following 400 ps and further decreased to 0 K in the last 400 ps. Ten structures were selected based on lowest energy and used for further analysis.

### DFT Geometry Optimization

DFT geometry optimization was performed with ORCA utilizing gCP geometrical counterpoise correction,^24^ D3BJ atom-pairwise dispersion correction with the Becke–Johnson damping scheme,^25,26^ the def2-mSVP basis set, def2/J auxiliary basis set,^27^ and employing the PBEh-3c composite approach.^28^ An implicit water model was included using the CPCM method.^29^ The ^oxo^G-quartet was first generated in Avogadro and converted to an ORCA input file. The ORCA output structures were analyzed using UCSF Chimera.^30^

## Results and Discussion

### ^oxo^G Analogues of d(TG_4_T) Form Parallel G-Quadruplexes

**Figure 1:**
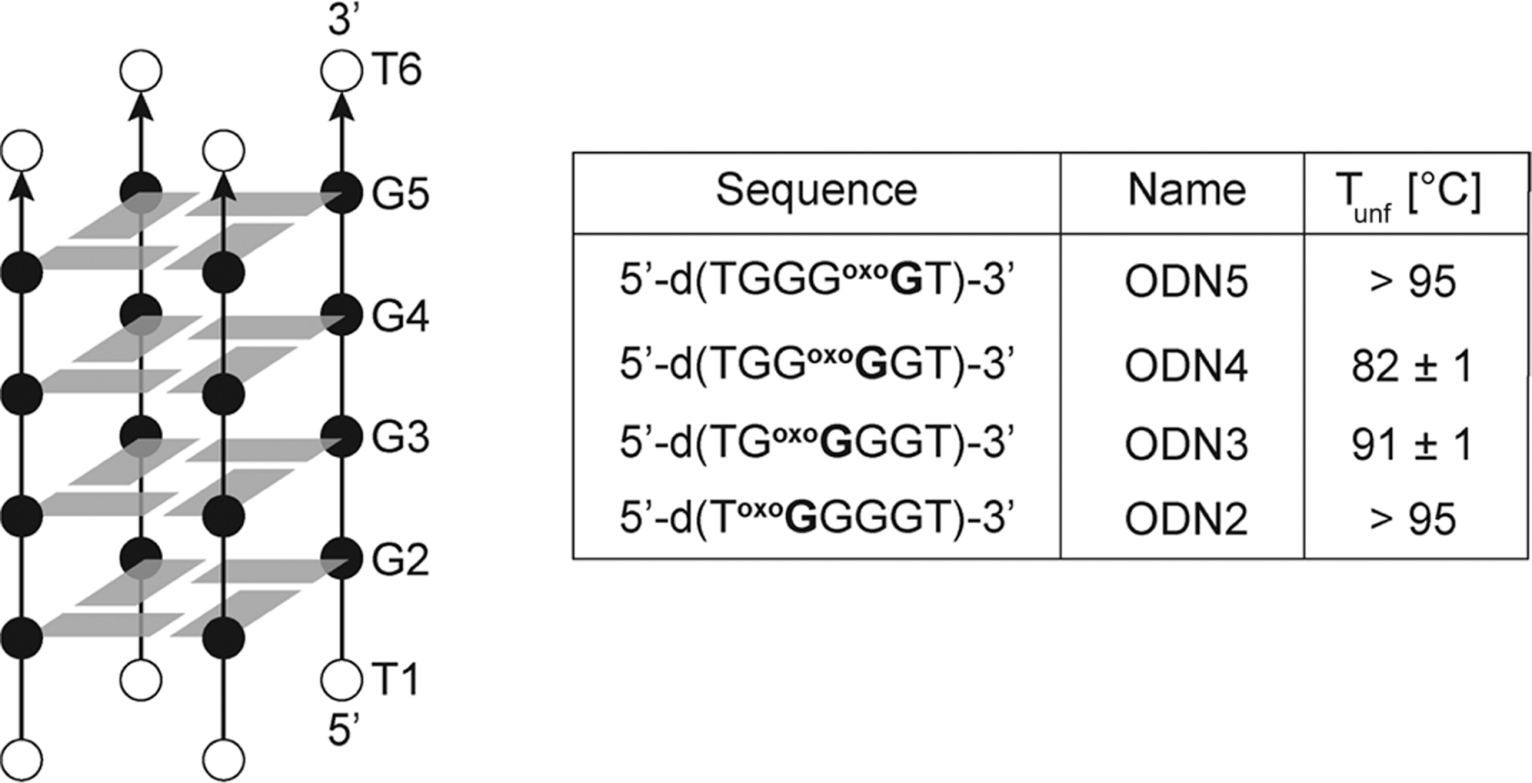
Schematic representation of the topology of the G-quadruplex adopted by d(TG_4_T). White spheres depict thymine nucleotides. Gray rectangles and black spheres are used to depict guanine nucleotides involved in G-quartets. Numbering of nucleotides is indicated along the right side of the scheme. Sequences, names used in this study, and temperatures of unfolding of ^oxo^G analogues are presented in the table. T_unf_ is the apparent midpoint of the thermal unfolding absorbance curve of a tetramolecular G-quadruplex. Oligonucleotides were dissolved in 50 mM KCl, 50 mM NaCl, 5 mM KPi, pH 7. The concentration of oligonucleotides was between 8 and 12 μM per strand.

**Figure 2:**
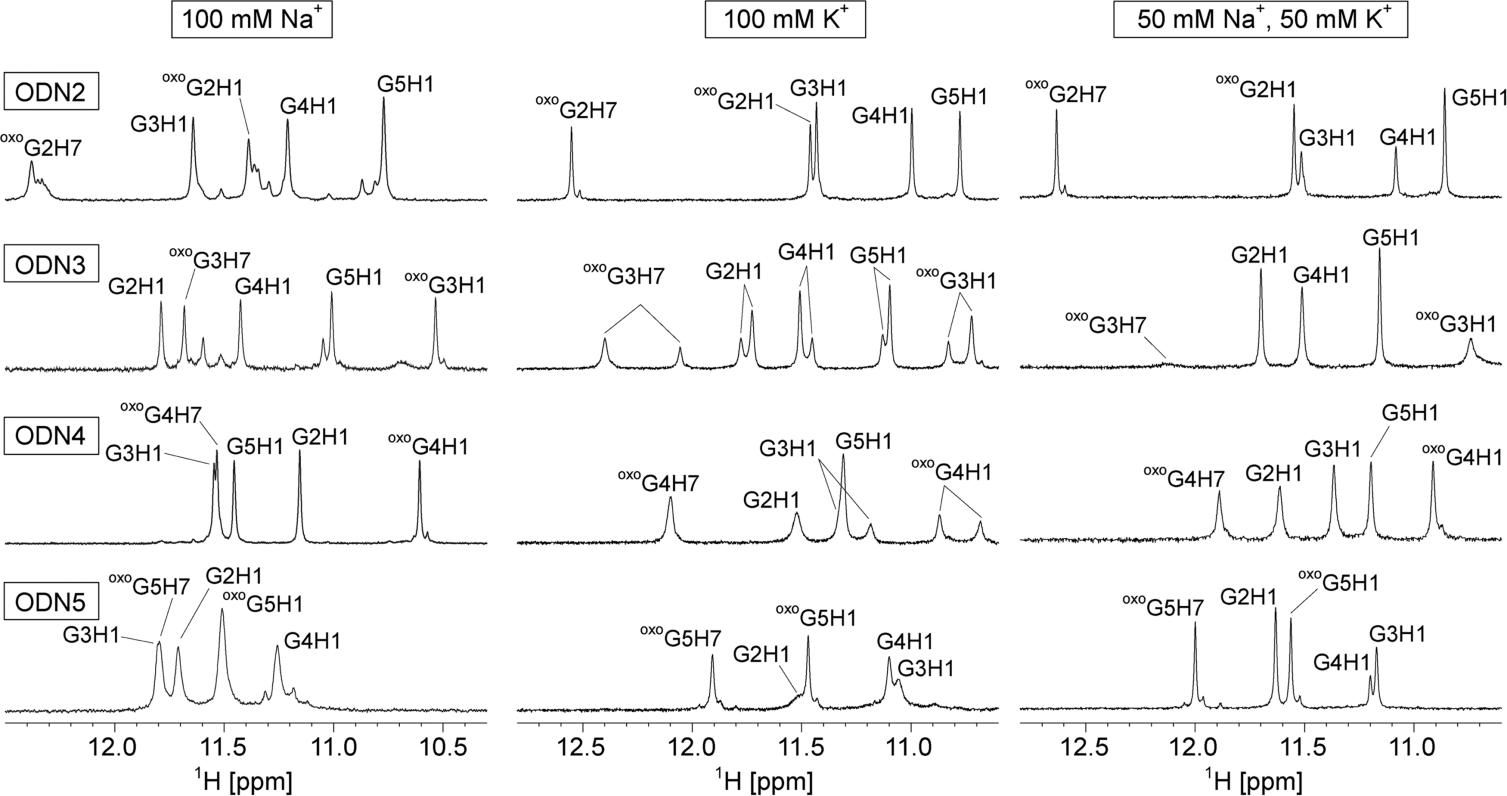
Imino regions of 1D ^1^H NMR spectra of ODN2-5 at 25 °C in the presence of 100 mM NaCl, 100 mM KCl, and a mixture of 50 mM NaCl and 50 mM KCl in 90%/10% H_2_O/^2^H_2_O. Solutions were buffered at pH 7 with 10 mM NaPi (in the case of Na^+^ cation solution), 10 mM KPi (in the case of K^+^ cation solution), or 5 mM KPi (in the case of mixed Na^+^ and K^+^ cation solution). The concentration of oligonucleotides ranged from 0.3 to 1.0 mM per strand. All spectra presented in this figure were recorded 48 h after addition of salt(s).

Analysis of imino regions of ^1^H NMR spectra characteristic for Hoogsteen hydrogen bonding of ^oxo^G analogues of d(TG_4_T) (designated as ODN2-5) reveals their folding into G-quadruplex structures in the presence of Na^+^ and/or K^+^ ions (Figures 1 and 2). Notably, an ^oxo^G nucleobase gives rise to a pair of imino resonances due to protons attached to both N1 and N7. H7 resonances of ^oxo^Gs are generally found downfield from the corresponding H1 resonances most likely due to the deshielding effect of the adjacent carbonyl group. Folding in the presence of 100 mM NaCl is slow and significant amounts of oligonucleotides remained unfolded even after several days of incubation at room temperature (Figure S1). On the other hand, folding of ODN2-5 in the presence of 100 mM KCl is fast (within minutes) (Figure S2). Interestingly, spectra of ODN3 and ODN4 in the presence of K^+^ cations give rise to two sets of some imino, aromatic and deoxyribose ^1^H resonances (Figures 2 and S2), which suggests that two major species coexist in solution and are involved in slow exchange on the NMR chemical shift timescale (Figure S3). At 25 °C the ratio of signal integrals between imino resonances of the two species in ODN3 is 1:2, while it is 1:1 in ODN4. However, at higher temperatures, the resolved sets of two resonances coalesce as the exchange between the two species is accelerated (Figure S4).

Dissolving ODN2-5 in a mixed salt solution of 50 mM NaCl and 50 mM KCl gives best NMR spectral quality with complete and fast folding into G-quadruplex structures. Furthermore, the split resonances in ODN3 and ODN4 coalesced (Figure 2). Therefore, we have attributed the signal splitting in ODN3 and ODN4 to two long-lived localization modes of K^+^ cations in the vicinity of ^oxo^G nucleotides. It is noteworthy that broadening of ^oxo^G3′s imino resonances is observed in spectra of ODN3 under these conditions. Interestingly, the effect of Na^+^ cations could not be replicated by adding NH_4_^+^ cations as no resolving of the split resonances was observed in ODN3 and ODN4 (Figure S5). Furthermore, resolving of the split resonances was also achieved with the addition of Cs^+^ cations to ODN3 and ODN4 G-quadruplexes in a K^+^ cation solution. Only imino resonances belonging to ^oxo^G protons show considerable chemical shift differences after addition of Cs^+^, indicating that Cs^+^ must localize in the vicinity of ^oxo^Gs. (Figure S6). Due to their favorable spectral properties, G-quadruplexes of ODN2-5 dissolved in a mixture of 50 mM NaCl and 50 mM KCl were chosen for further characterization. Low-intensity imino resonances (<5%) were also noted in NMR spectra of ODN2-5, but could not be characterized in detail due to the low population of these minor species.

The stability of G-quadruplexes was evaluated with UV melting experiments of ODN2-5 in a mixture of 50 mM NaCl and 50 mM KCl (Figures 1 and S7). The UV melting profile of ODN2 does not exhibit signs of unfolding at up to 95 °C. Similarly, only initial indications of unfolding can be observed close to 95 °C for ODN5. This is consistent with the high thermal stability of the parent d(TG_4_T)_4_ G-quadruplex, which showed no signs of unfolding up to 90 °C in a 110 mM KCl solution.^15^ On the other hand, the thermal stabilities of ODN3 and ODN4 are lower with T_unf_ values of 91 and 82 °C, respectively.

**Figure 3:**
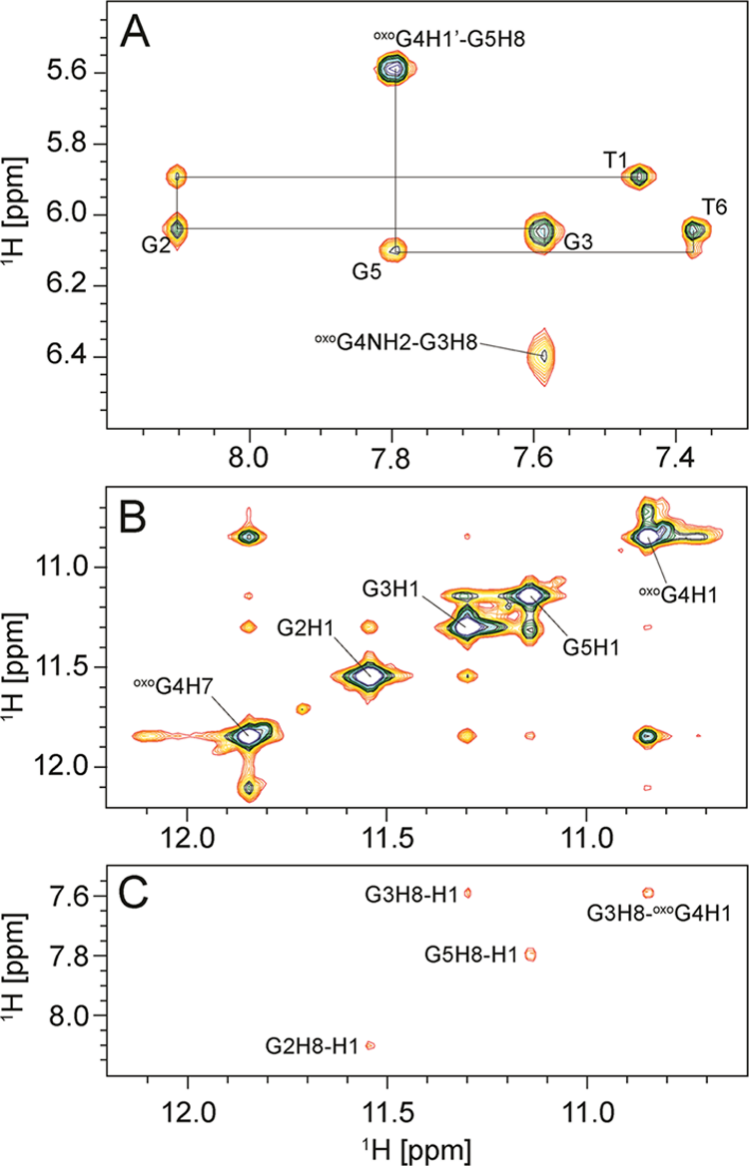
Selected regions of a NOESY spectrum of ODN4. (A) Aromatic-anomeric, (B) imino-imino, and (C) imino-aromatic regions of a NOESY spectrum (τ_m_ = 250 ms) in the presence of 50 mM NaCl and 50 mM KCl, 5 mM KPi, pH 7, 90/10% H_2_O/^2^H_2_O. The concentration of ODN4 was 0.3 mM per strand. The sequential walk, interrupted between G3 and ^oxo^G4, is depicted in (A) with intra-nucleotide cross-peaks annotated.

A detailed structural analysis of G-quadruplexes adopted by ODN2-5 was undertaken with a set of NMR experiments. Imino, aromatic, methyl, and well-resolved deoxyribose proton resonances in NOESY spectra of ODN2-5 in the presence of 100 mM NaCl, 100 mM KCl, and a mixture of 50 mM NaCl and 50 mM KCl were assigned (Figure 3). Sequential walks can be traced in aromatic-anomeric regions of NOESY spectra for ODN2-5 regardless of the type of cations present in solution and exhibit similar connectivity patterns suggesting that all G-quadruplexes adopt the same topology. Furthermore, the two major species observed in solutions of ODN3 and ODN4 in the presence of K^+^ cations exhibit identical NOE cross-peak patterns, which is in agreement with our rationale that the two species differ only in K^+^ cation localization.

Intensities of intra-residual cross-peaks in NOESY spectra of ODN2-5 correspond to all G and T nucleotides adopting the *anti* conformation, which is typical for parallel G-quadruplexes (data for ODN4 is shown as an example in Figure 3). As expected, sequential walks for ODN2-5 are interrupted at ^oxo^G positions due to absence of aromatic H8 protons. Consequently, we were not able to directly determine the preference for *syn* or *anti* conformations of ^oxo^G based on the intensity of ^oxo^G_(i)_H1′-H8 cross-peaks in NOESY spectra alone. Therefore, we resorted to the analysis of chemical shifts of H2′ and C2′ nuclei (Figures S8 and S9). In ODN2-5, H2′ resonances of ^oxo^Gs are shifted downfield (δ 3.1–3.5 ppm) compared to Gs (δ 2.2–2.6 ppm). Furthermore, C2′ resonances of ^oxo^Gs are shifted upfield (δ 32.3–35.3 ppm) compared to Gs (δ 37.5–39.8 ppm). This is in agreement with chemical shifts for 8-substituted purine nucleosides in the *syn* conformation,^31^ and suggests that all ^oxo^G nucleotides in ODN2-5 are in the *syn* conformation. Interestingly, intense cross-peaks can be observed in NOESY spectra of ODN2-5 between anomeric protons of ^oxo^Gs and aromatic protons of subsequent nucleotides [i.e., ^oxo^G_(i)_H1′-G_(i+1)_H8] (Figure 3A). Furthermore, amino protons of ^oxo^Gs are found to be isochronous at 25 °C and with their chemical shifts in the range from δ 6.1 to 6.5 ppm (Figure S10), suggesting they are not involved in hydrogen bonds.

In the imino-imino regions of NOESY spectra only sequential connectivities are observed (Figure 3B) and the imino-aromatic regions exhibit G_(i)_H1-G_(i)_H8 cross-peaks (Figure 3C), both of which are in agreement with the parallel G-quadruplex topology. Additionally, ^oxo^G_(i)_NH_2_-G_(i–1)_H8 (Figure 3A) and ^oxo^G_(i)_H1-G_(i–1)_H8 (Figure 3C) cross-peaks are observed in NOESY spectra of ODN2-5. However, due to the symmetry of ODN2-5 G-quadruplexes, it is ambiguous if these cross-peaks are of intra- or interstrand nature.

### ^oxo^G nucleobases Exhibit a Distinct Intra-Quartet Hydrogen-Bonding Scheme

**Figure 4:**
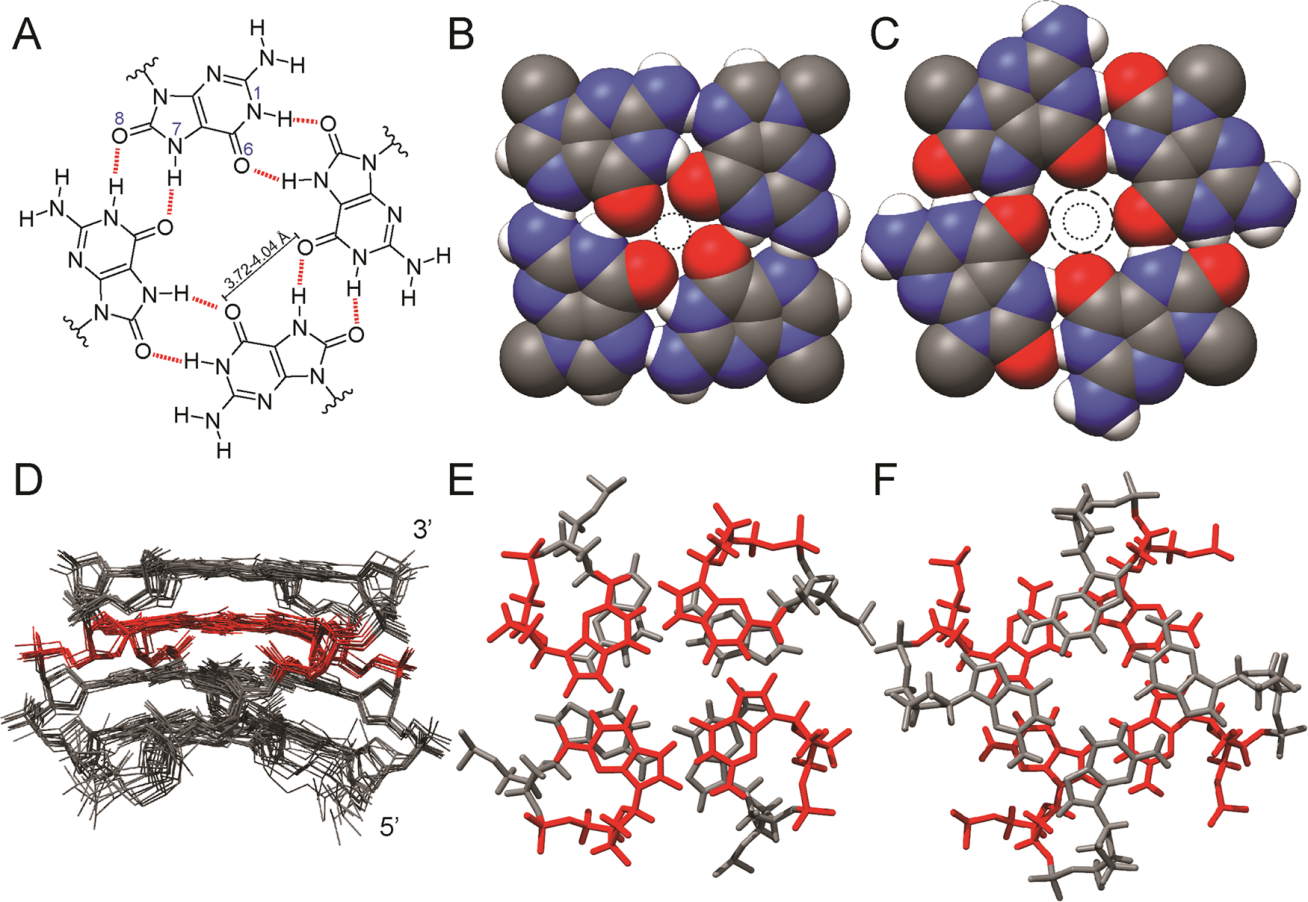
Structural insights into ^oxo^G-quartets. (A) Schematic presentation of an ^oxo^G-quartet with hydrogen bonds shown as red dashed lines, distance between neighboring O6 atoms and numbering of oxygen and nitrogen atoms, participating in hydrogen bonding. Space-filling model of (B) a G-quartet (PDB ID: 352D) and (C) DFT-optimized quartet of 9-methyl-8-oxoguanines. A comparison of the maximum diameter of a sphere that fits the cavities of the G-quartet (diameter of 1.46 Å) and ^oxo^G-quartet (diameter of 2.68 Å) is shown in (B) and (C), respectively. (D) Superposition of ten lowest-energy structures of ODN4 obtained with simulated annealing. Thymine nucleotides are omitted for clarity. Stacking of an ^oxo^G-quartet (red) with adjacent (E) 5′ or (F) 3′ G-quartets (gray) in the lowest-energy structure of ODN4.

The hydrogen-bonding network of ^oxo^G nucleotides within G-quadruplex structures was determined via analysis of NOE connectivities. Upfield chemical shifts of amino protons of ^oxo^Gs (*vide supra*) suggest that they are not hydrogen-bond donors. On the other hand, downfield shifted narrow H1 and H7 resonances of ^oxo^G suggest that both protons are protected from exchange with bulk solvent and involved in hydrogen bonds. We observe intense NOE cross-peaks between H1 and H7 of ^oxo^Gs (Figure 3B). Such cross-peaks cannot arise from intra-nucleotide correlations due to the large distance between H1 and H7 within the ^oxo^G nucleobase (cca. 5.0 Å). ^oxo^Gs also cannot be positioned in different G-quartet planes as this would result in a minimum plane separation distance of 3.4 Å. Furthermore, ^oxo^Gs in different quartet planes is not in agreement with the NMR data, which shows the formation of symmetrical parallel G-quadruplexes. However, a simple model with a planar arrangement of four ^oxo^G nucleobases exhibits short H1 to H7 distances (cca. 2.3 Å), which is in agreement with collected NMR data. Their interpretation led us to propose the formation of an ^oxo^G-quartet, comprised of four ^oxo^G nucleobases, connected via hydrogen bonds N1–H1···O8 and N7–H7···O6 (Figure 4A). The same hydrogen-bonding arrangement was already proposed for helix-forming lipophilic 8-oxoguanine derivatives.^32^

A detailed model of an ^oxo^G-quartet, formed by four 9-methyl-8-oxoguanines, was obtained via DFT optimization (using the def2-mSVP basis set and PBEh-3c method). Simulations were carried out without cations and with either a Na^+^ or a K^+^ cation in-plane and in-line with the ion cavity of the ^oxo^G-quartet. During the optimization planarity of the ^oxo^G-quartet was constrained to prevent any out-of-plane movement of the nucleobases. Selected distances in the energy-optimized ^oxo^G-quartet geometries are summarized in Table 1. To assess the size of the central cavity of the ^oxo^G-quartet, we measured the distances between carbonyl group O6 atoms of the neighboring 9-methyl-8-oxoguanines (Figure 4A). The distance ranges from 3.72 Å, when a Na^+^ cation is positioned in the plane of the ^oxo^G-quartet, to 4.04 Å, when no cation is present.

**Table 1:** Structural Details of DFT-Optimized ^oxo^G-Quartets

	No M^+^ (Å)	K^+^ (Å)	Na^+^ (Å)
N1–H1···O8^a^	1.92	1.91	1.88
N7–H7···O6^a^	1.76	1.75	1.74
N1···O8^b^	2.95	2.93	2.90
N7···O6^b^	2.78	2.78	2.76
neighboring O6–O6^c^	4.04	3.87	3.72
diagonal O6–O6^d^	5.72	5.47	5.27

a Distances in DFT-optimized ^oxo^G-quartets without cations and with a Na^+^ or K^+^ cation positioned in the center of the ^oxo^G-quartet plane. DFT optimization was done using the PBEh-3c method and the def2-mSVP basis set. Distance between hydrogen atom and hydrogen-bond acceptor.

b Distance between hydrogen-bond donor and hydrogen-bond acceptor.

c Distance between the O6 atoms of two neighboring 9-methyl-8-oxoguanines.

d Distance between the O6 atoms of the two diagonally opposite 9-methyl-8-oxoguanines.

The distance between diagonally opposite O6 atoms in the ^oxo^G-quartet ranges from 5.27 to 5.72 Å. In comparison, the average distance between diagonally opposite O6 atoms in G-quartets (PDB: 352D(33)) amounts to 4.50 Å. Taking into account the van der Waals radii of the O6 atoms, a sphere with a maximum diameter of 2.68 and 1.46 Å is able to fit into the ^oxo^G-quartet and G-quartet cavity, respectively (Figure 4B,C). Hydrogen-bond distances are less variable between the optimized geometries, with a maximum difference between the distances of the N1–H1···O8 hydrogen bond amounting to 0.04 Å. A comparison of space-filling models of a G-quartet (PDB: 352D(33)) and a DFT-optimized ^oxo^G-quartet is shown in Figure 4, demonstrating the larger central cavity of the ^oxo^G-quartet.

Using the optimized geometry of the ^oxo^G-quartet, we have performed atomistic simulations of ODN2-5 G-quadruplexes using a simulated annealing protocol. Since ODN2-5 G-quadruplexes exhibit fourfold symmetry, we were unable to unambiguously determine inter- or intra-nucleotide nature of certain NMR distance constraints. Therefore, we only included hydrogen-bond distance and glycosidic torsion angle restraints in simulated annealing calculations. Good convergence was achieved over 100 runs of simulated annealing (Figure 4D and Table S1). Calculations resulted in parallel, right-handed G-quadruplex structures, with G and ^oxo^G nucleotides in *anti* and *syn* conformations, respectively. Efficient stacking of six-membered rings of ^oxo^G_(i)_ and G_(i–1)_ nucleobases is observed in all structures (Figure 4E and Table 2). On the other hand, only partial stacking of five-membered rings can be observed between ^oxo^G_(i)_ and G_(i+1)_ nucleobases, resulting in a much lower stacking surface (Figure 4F and Table 2). ^oxo^G has an effect on the rise and twist, with the ^oxo^G_(i)_-G_(i+1)_ step exhibiting a larger rise parameter compared to the rest of the structure and the twist being considerably larger for the steps which include the ^oxo^G nucleotide (Table 2).

**Table 2:** Three Different Nucleotide Steps in ODN2-5

	stacking surface^a^ [Å^2^]	rise [Å]	twist [°]
G_(i–1)_-^oxo^G_(i)_	6.79 ± 1.52	2.94 ± 0.23	26.23 ± 6.69
^oxo^G_(i)_-G_(i+1)_	1.59 ± 1.17	3.21 ± 0.31	26.52 ± 5.37
G-G^b^	4.22 ± 1.62	2.97 ± 0.14	21.95 ± 4.60

a Average rise, twist and stacking surface with standard deviation in the 10 lowest-energy MD structures. Stacking surface includes exocyclic atoms.

b Average of all G–G steps in ODN2-5 structures.

**Figure 5:**
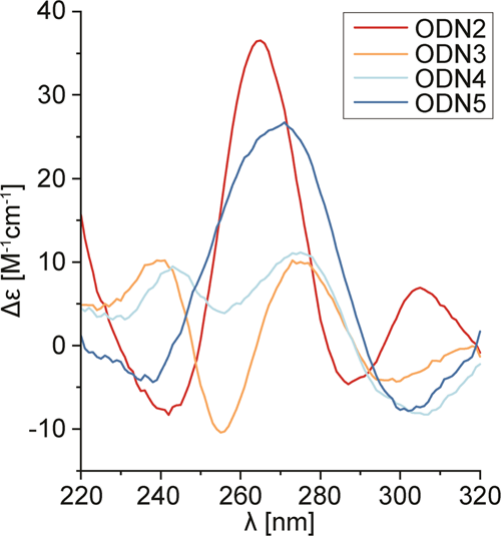
CD spectra of ODN2-5 in the presence of 50 mM NaCl, 50 mM KCl, and 5 mM KPi, pH 7. DNA concentrations were 0.5 mM per strand.

Specific stacking of ^oxo^G nucleobases is reflected in CD spectra of ODN2-5 (Figure 5). ODN2 and ODN5 with terminal ^oxo^G-quartets exhibit CD maxima at 265 and 270 nm, respectively. On the other hand, CD spectra of ODN3 and ODN4, with ^oxo^G-quartets sandwiched between G-quartets, exhibit two maxima at 240 and 275 nm. Interestingly, a shoulder is present in the CD spectrum of ODN2 above 300 nm, which may point to DNA condensation into higher-ordered structures.^34^

Oxidation of G to ^oxo^G leads to a rearrangement of hydrogen-bond donors and acceptors on the Hoogsteen edge of the nucleobase. The newly protonated N7 precludes the formation of the N2–H2···N7 hydrogen bond found in canonical G-quartets. Our previous study showed that a single ^oxo^G lesion in a G-quartet can be tolerated, with the O6 of ^oxo^G serving as a hydrogen-bond acceptor for a bifurcated hydrogen bond with the neighboring guanine.^13^ Furthermore, G-quartet formation was not hindered when an ^oxo^G was hydrogen-bonded with a neighboring xanthine.^17^ Here, we show that an ^oxo^G-quartet can also be a stable structural element within the G-quadruplex core with H7 as a hydrogen-bond donor.

While the planar ^oxo^G-quartet stacks well with an adjacent 5′ G-quartet, stacking with a 3′ G-quartet is less efficient, as conferred through analysis of stacking surfaces. Nevertheless, G-quadruplexes with 5′- and 3′-terminal ^oxo^G-quartets in ODN2 and ODN5, respectively, are more thermally stable than ODN3 and ODN4 with an ^oxo^G-quartet sandwiched between two G-quartets. This is also reflected in differences in CD spectra of ODN2 and ODN5 versus ODN3 and ODN4 and is likely related to less constrained positioning of ^oxo^G at 5′- and 3′-termini of the G-quadruplex core.^35^

Since the *anti* conformation is unfavorable for nucleotides with a (bulky) substituent at position 8 (e.g., ^oxo^G), it is not surprising that the ^oxo^G-quartet features an all-*syn* arrangement. Interestingly, d(TG_4_T)_4_ was reported to exhibit 15% of 5′-end G-quartets in all-*syn* orientation.^36^ A study has shown that a slow dynamic interconversion between all-*syn* and all-*anti* G-quartets is possible without disrupting the whole G-quadruplex core.^37^ This is not the case here with ODN2-5 where no *syn-anti* flipping of nucleobases could be detected. However, perturbations in the hydrogen-bond network cause mutual repositioning of ^oxo^G nucleobases. Using DFT optimization, we showed that ^oxo^G-quartets exhibit a central cavity, with a 4.04 Å distance between O6 atoms of two neighboring 9-methyl-8-oxoguanines. For comparison, the average O6–O6 distance in G-quartets is 3.15 Å, which makes the central cavity in ^oxo^G-quartets considerably larger. Studies of crystal structures showed that K^+^ cations localize equidistantly between two G-quartet planes.^38,39^ On the other hand, due to their smaller ionic radius, Na^+^ cations can localize in a G-quartet plane or any distance between two planes.^33^ However, due to the larger cavity in the center of an ^oxo^G-quartet and only minor differences in H-bond lengths and neighboring carbonyl distances the absence or presence of different cations, an in-plane localization of Na^+^ as well as K^+^ cations is feasible. Furthermore, due to reduced steric restrictions cation movement through the ^oxo^G-quartet plane is expected to be faster (i.e., exchange between binding sites).

**Figure 6:**
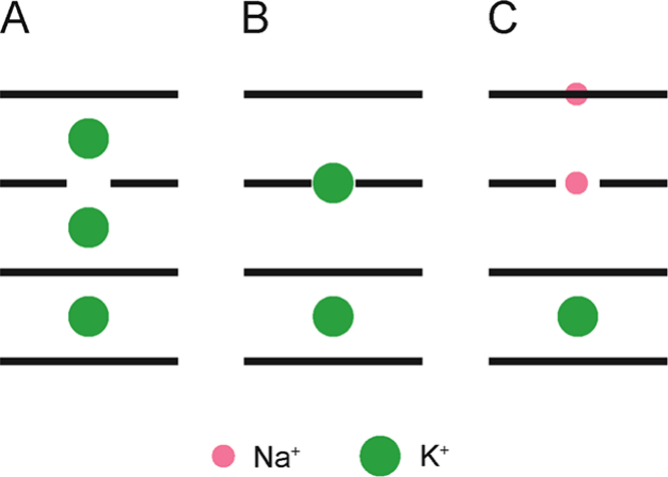
Proposed localization of Na^+^ and K^+^ cations within the ODN4 G-quadruplex. K^+^ cation may localize in between the quartet planes (A) or in an ^oxo^G-quartet plane (B). Mixed Na^+^/K^+^ form (C) could exhibit in-plane bound Na^+^ cations. Unbroken and broken lines represent G-quartets and ^oxo^G-quartets, respectively.

We have observed that in the presence of K^+^ cations alone, two structures were present in solution for ODN3 and ODN4 at 25 °C, which were shown with 2D ROESY spectra to be in slow exchange (Figure S3). At 25 °C, the maximum chemical shift difference between a pair of doubled resonances (^oxo^G3H7 in ODN3) at a magnetic field of an 800 MHz spectrometer is 0.34 ppm, which corresponds to a lifetime of 3.6 ms. We have eliminated the possibility of switching of the glycosidic conformation or sugar-repuckering of ^oxo^G nucleotides, since no relevant cross-peaks could be observed in NOESY or DQF-COSY/TOCSY experiments. We have also eliminated the possibility of tautomerism of the 8-oxoguanine moiety. Chemical shifts and intensities of the imino proton resonances are pH-independent, which does not support the formation of an 8-hydroxy tautomer (Figure S11). In full agreement, quantum mechanical studies showed that the 8-keto tautomer is predominant.^40^ Instead, we propose that the two species of ODN3 and ODN4 differ in K^+^ cation localization. This is in agreement with the millisecond lifetimes of ammonium ions bound within the d(TG_4_T) G-quadruplex.^23^ K^+^ cations may localize either between an ^oxo^G and a G-quartet or in an ^oxo^G-quartet plane (Figure 6). Due to mutual Coulombic repulsion, one K^+^ cation could be ejected from the G-quadruplex structure. Nevertheless, in-plane K^+^ localization appears to be suboptimal since the addition of smaller size Na^+^ cations to existing solutions of ODN3 and ODN4 with K^+^ leads to resolution of doubled resonances in ^1^H spectra. The addition of Na^+^ cations likely eliminates K^+^ movement by preferential localization of Na^+^ in-plane of ^oxo^G-quartets. Interestingly, the same effect was observed with the addition of Cs^+^ cations. However, binding of the considerably larger (with respect to Na^+^ and K^+^) Cs^+^ cation is expected between a G-quartet and ^oxo^G-quartet plane (*vide supra*). In ODN2 and ODN5, where ^oxo^G-quartets are positioned at the 5′- and 3′-termini of the G-quadruplex core, K^+^ cation movement is fast at temperatures as low as 5 °C and only single sets of NMR resonances can be observed (Figure S12). NMR spectra of ODN2 and ODN5 did not show any significant differences after the addition of Na^+^ cations, except for minor changes in imino proton chemical shifts and resonance linewidths (Figure 2).

Based on the results reported herein and on previous studies of the effect of ^oxo^G incorporation on the structure and stability of G-quadruplexes,^13,21^^oxo^G may act as a cryptic lesion in the context of G-rich regions without the presence of a complementary strand. Proposed pathways of ^oxo^G repair in double-stranded G-rich DNA also stipulate that ^oxo^G is not destabilizing enough to promote a duplex-to-quadruplex transition. Destabilization of the duplex and transition to a G-quadruplex is thought to occur after excision of ^oxo^G via the action of the glycosylase OGG1, which yields an abasic site.^41,42^

Oxidative damage *in vivo* is highly unlikely to introduce more than a single ^oxo^G lesion within short DNA stretches needed for one G-quadruplex unit.^43^ However, the d(TG_4_T)_4_ model system demonstrates how redistribution of hydrogen-bond donors and acceptors affects the structure of nucleic acids, which can also have a functional effect. For instance, siphoviruses use aminoadenine instead of adenine in their genome, therefore having three hydrogen bonds in the aminoadenine–thymine base pair, and have a DNA polymerase that preferentially selects aminoadenine for a thymine template.^44^ Since ^oxo^G is a DNA lesion frequently occurring alongside pathophysiological changes,^45,46^ insights into the effect of guanine oxidation on structural changes of nucleic acids in functionally important genome regions could reveal possible disease origins.

## Supporting Information Available

Additional 1D and 2D NMR spectra, UV melting curves, and lowest-energy structures RMSD values (PDF)
