# Supplementary material for: 8-Oxoguanine Forms Quartets with a Large Central Cavity
Source: Biochemistry. 2022 Oct 19;61(21):2390–7. doi: 10.1021/acs.biochem.2c00478 (PMC9631995; doi:10.1021/acs.biochem.2c00478)
Supplement: Supplementary file 1 — bi2c00478_si_001.pdf [file bi2c00478_si_001.pdf]

Supplementary Information

8-Oxoguanine forms quartets with a large central cavity

Simon Aleksič[a,b], Peter Podbevšek[a] and Janez Plavec*[a,b,c]

[a] Slovenian NMR centre, National Institute of Chemistry, Hajdrihova 19, 1000 Ljubljana (Slovenia)

[b] Faculty of Chemistry and Chemical Technology, University of Ljubljana, Večna pot 113, 1000 Ljubljana (Slovenia)

[c] EN-FIST Centre of Excellence, Trg OF 13, 1000 Ljubljana (Slovenia)

Results

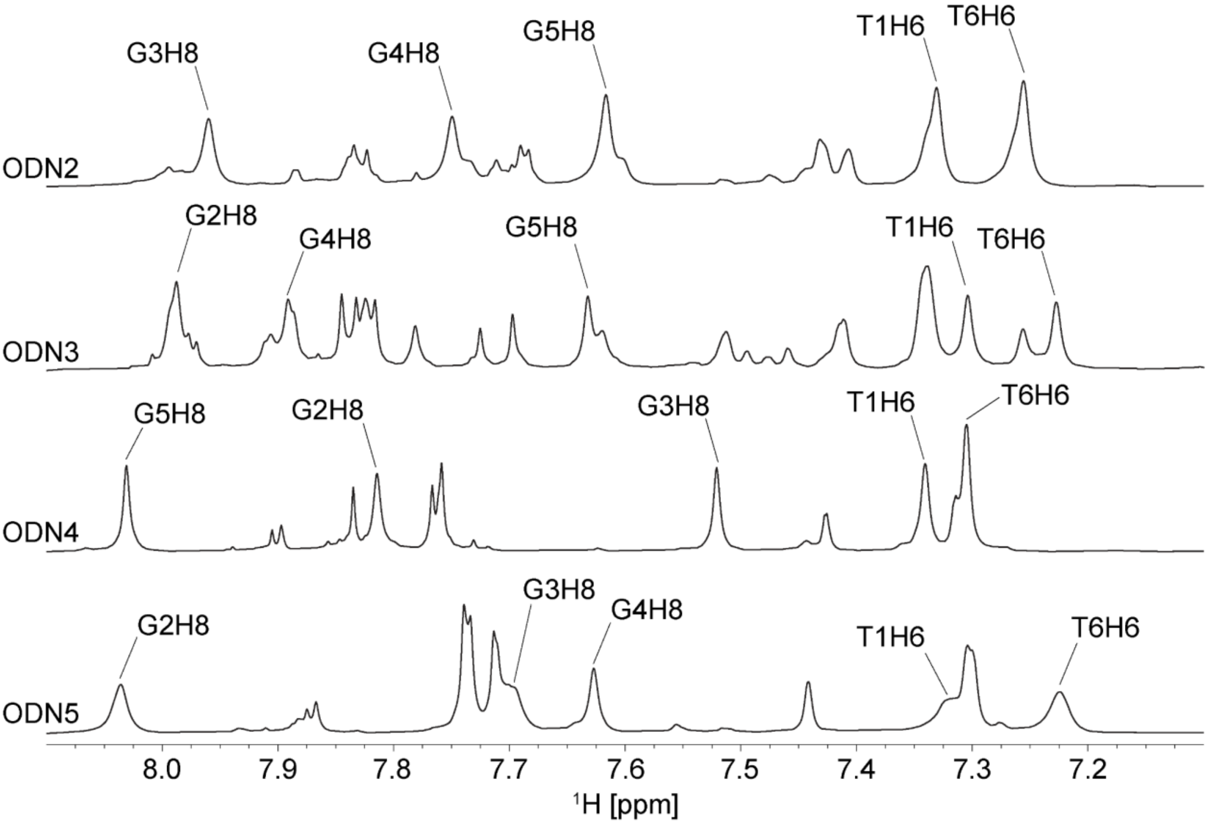

Figure S1: Aromatic regions of 1D ^{1}H spectra of ^{oxo}G analogues in a Na^{+} solution. Peaks belonging to aromatic protons of the G-quadruplex fold are labeled. Spectra were recorded at 25 °C on an 800 MHz spectrometer. Oligonucleotides were diluted in 100 mM NaCl, 10 mM NaPi, pH 7 and 90%/10% $^{1}H_2O/^{2}H_2O$. Concentration of oligonucleotides ranged from 0.7 to 1.0 mM per strand.

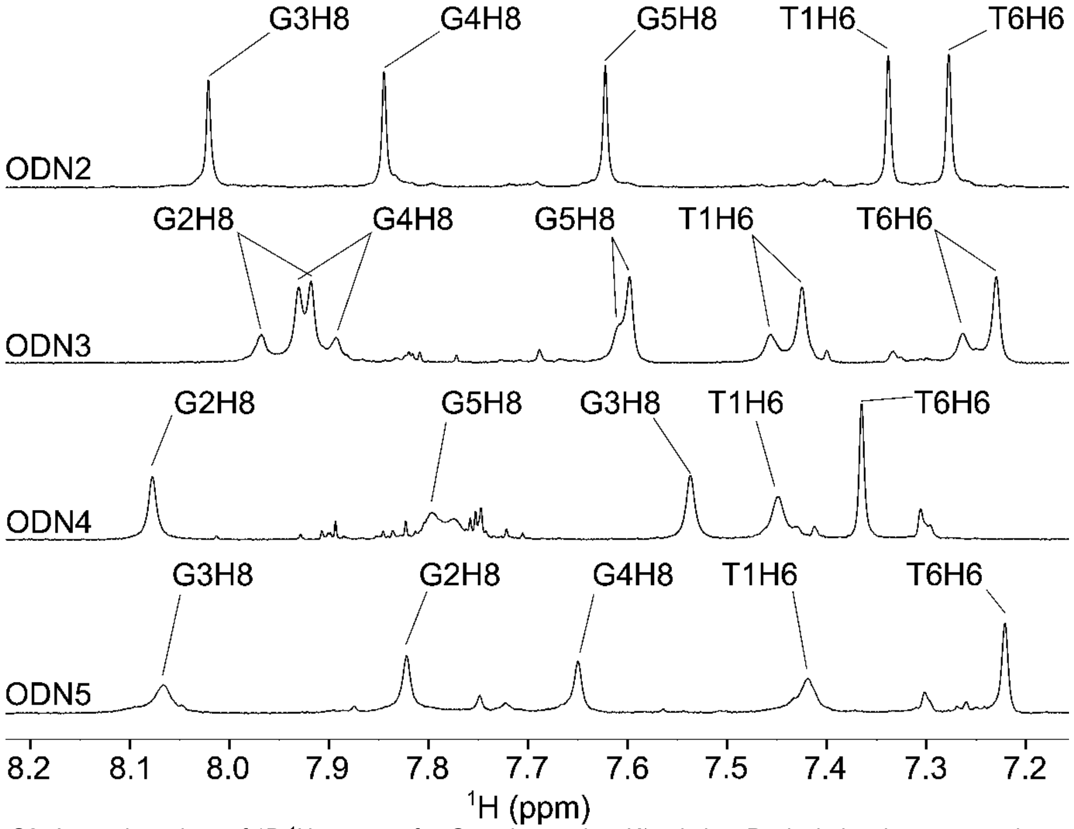

Figure S2. Aromatic regions of 1D ^{1}H spectra of ^{oxo}G analogues in a K^+ solution. Peaks belonging to aromatic protons of the G-quadruplex fold are labeled. Spectra were recorded at 25 °C on an 800 MHz NMR spectrometer. Oligonucleotides were diluted in 100 mM KCl, 10 mM KPi, pH 7 and 90%/10% $^{1}H_2O/^{2}H_2O$. Concentration of oligonucleotides ranged from 0.7 to 1.0 mM per strand.

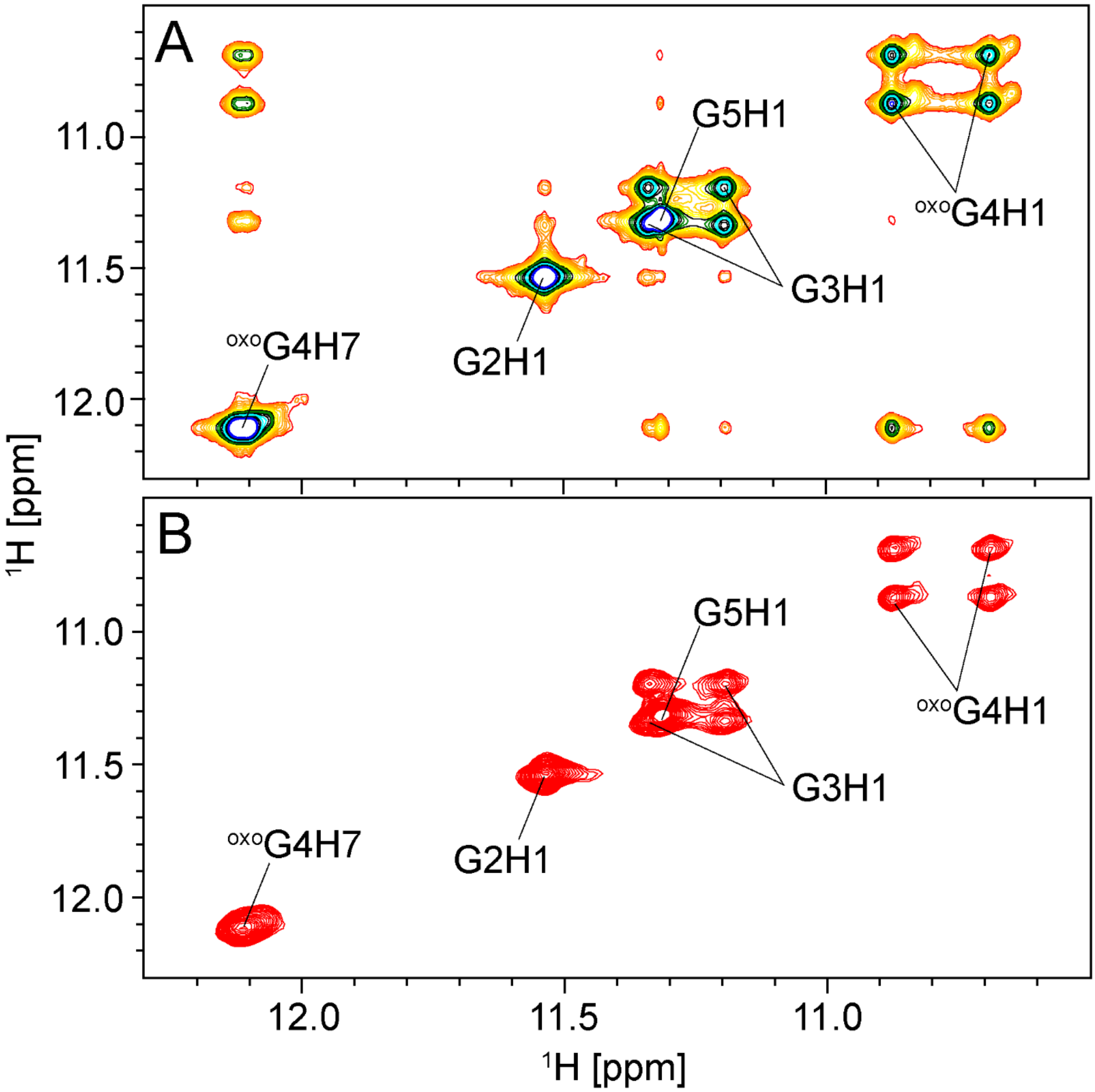

Figure S3. Imino-imino region of A) NOESY (τ_m = 250 ms, 25 °C) and B) ROESY (τ_m = 200 ms, 25 °C) spectra of ODN4 in 100 mM KCl, 10 mM KPi, pH 7 and 90%/10% $^1H_2O/^2H_2O$. Concentration of oligonucleotide was 0.7 mM per strand.

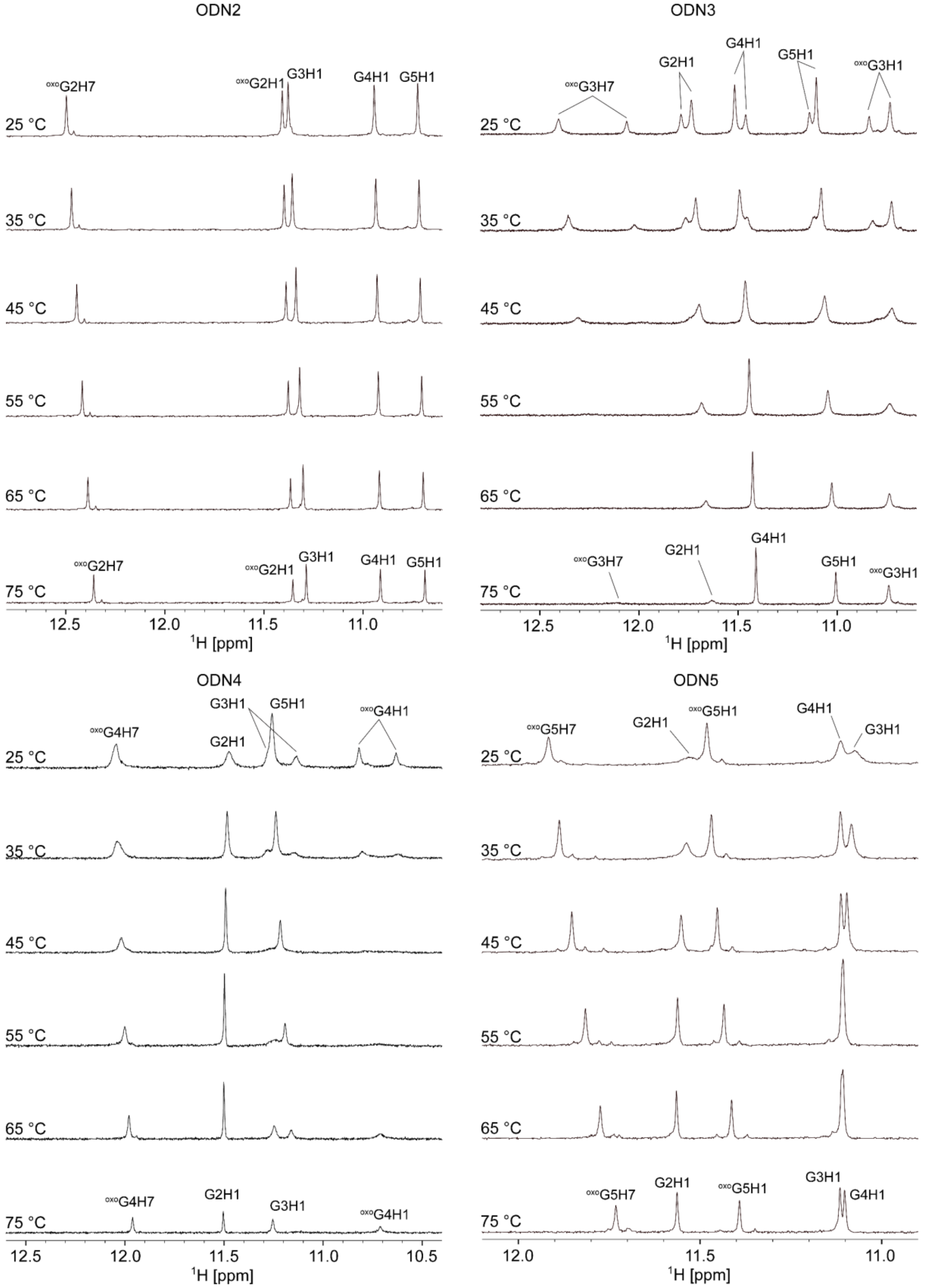

Figure S4. Imino region of ^{1}H 1D NMR spectra of ODN2-5 at temperatures from 25 to 75 °C. Spectra were recorded on an 800 MHz spectrometer. Oligonucleotides were diluted in 100 mM KCl, 10 mM KPi, pH 7 and 90%/10% $^{1}H_2O/^{2}H_2O$. Concentration of oligonucleotides ranged from 0.7 to 1.0 mM per strand.

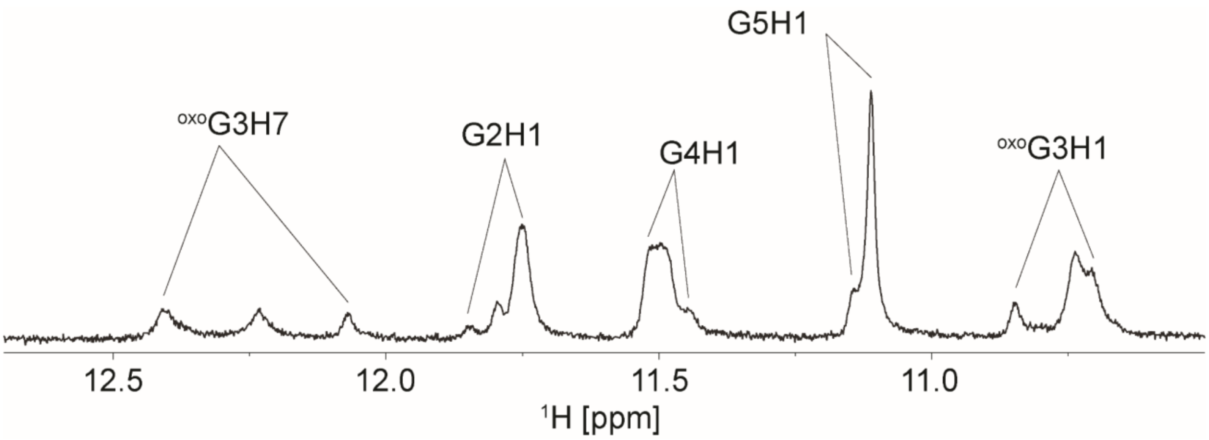

Figure S5. Imino region of ^{1}H 1D NMR spectrum of ODN3 at 25 °C. Spectrum was recorded on a 600 MHZ spectrometer. Oligonucleotide was dissolved in 50 mM KCl, 50 mM NH_4Cl, 5 mM KPi, pH 7, 90%/10% $^{1}H_2O/^{2}H_2O$. Concentration of oligonucleotide was 0.3 mM per strand.

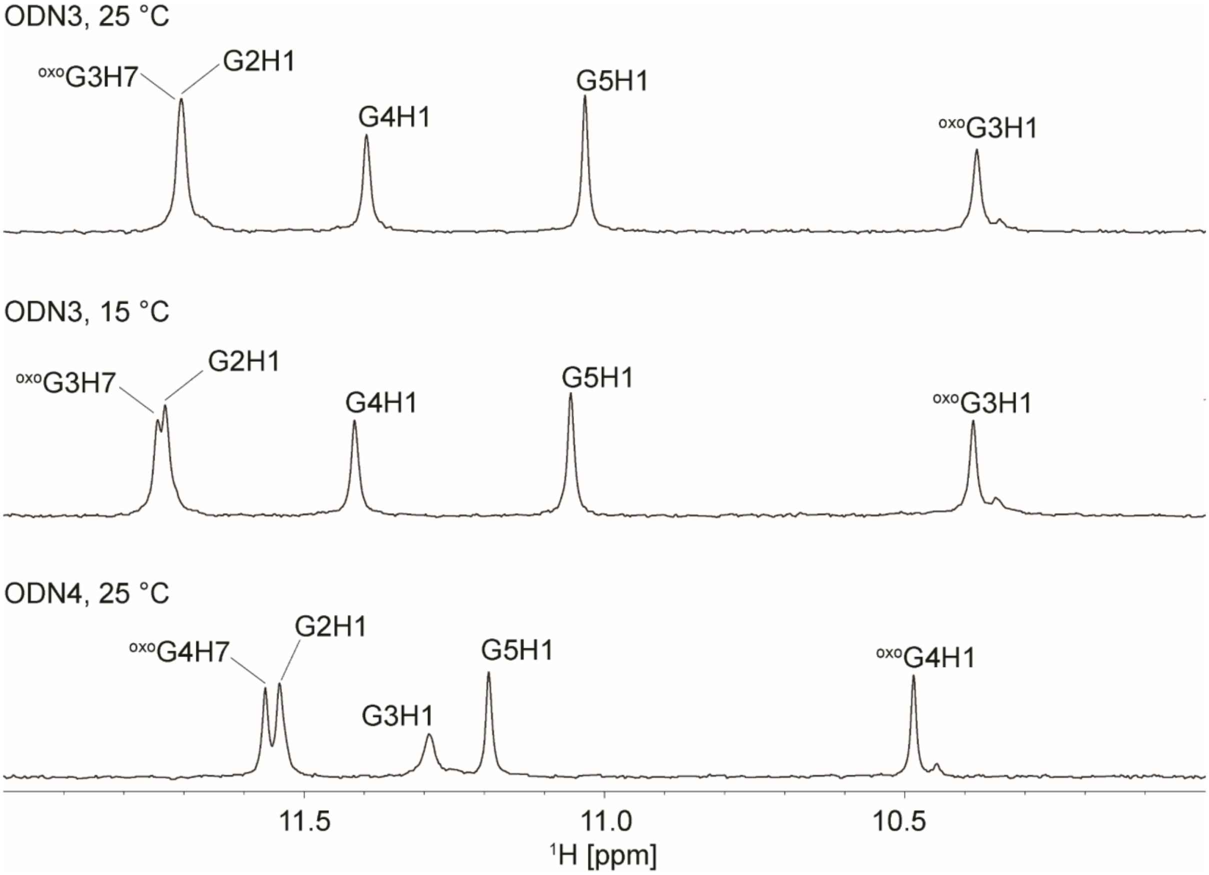

Figure S6. Imino region of ^{1}H 1D NMR spectra of ODN3 and ODN4 in a mixed K^+ and Cs^+ cation solution. Oligonucleotides were diluted in 50 mM KCl, 50 mM CsCl, 5 mM KPi, pH 7, 90%/10% $^{1}H_2O/^{2}H_2O$. Spectra were recorded on an 800 MHz NMR spectrometer at 25 and 15 °C. Oligonucleotide concentration ranged from 0.3 to 0.5 mM per strand.

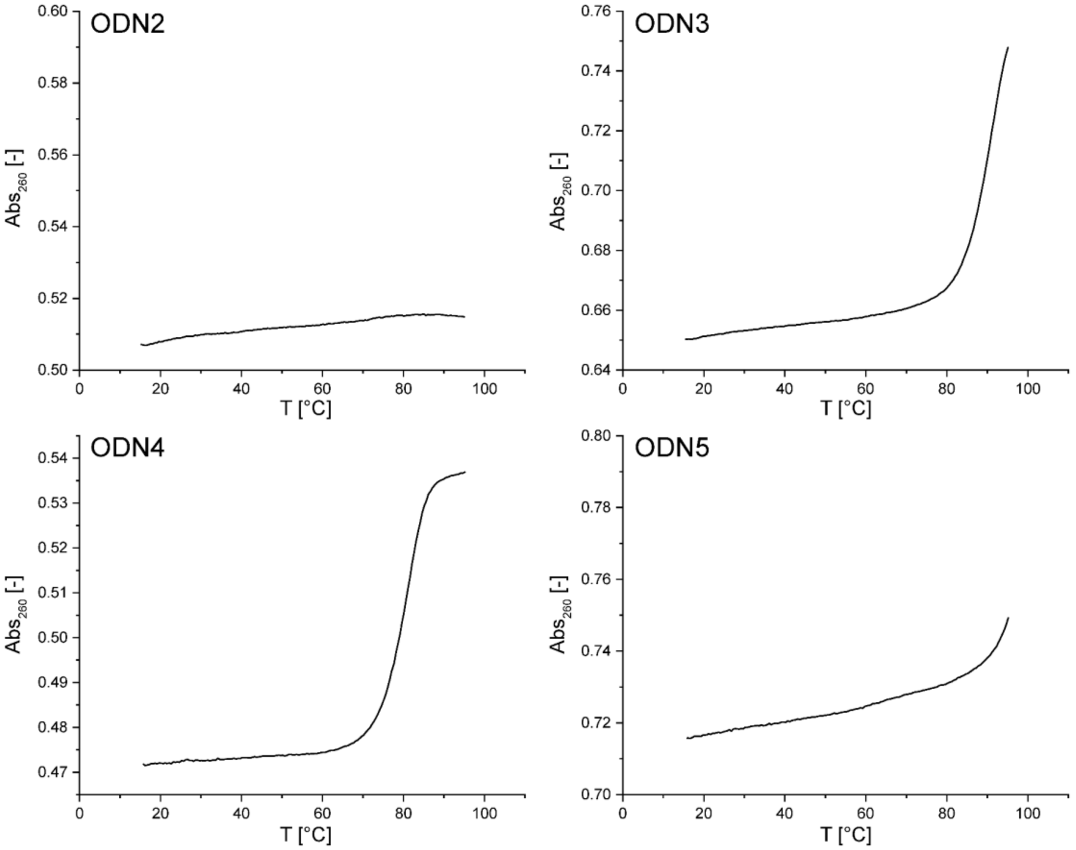

Figure S7: UV melting profiles of ODN2-5 at 260 nm. Oligonucleotides were diluted in 50 mM KCl, 50 mM NaCl, 5 mM KPi, pH 7. Concentration of oligonucleotides ranged from 8 to 12 µM per strand.

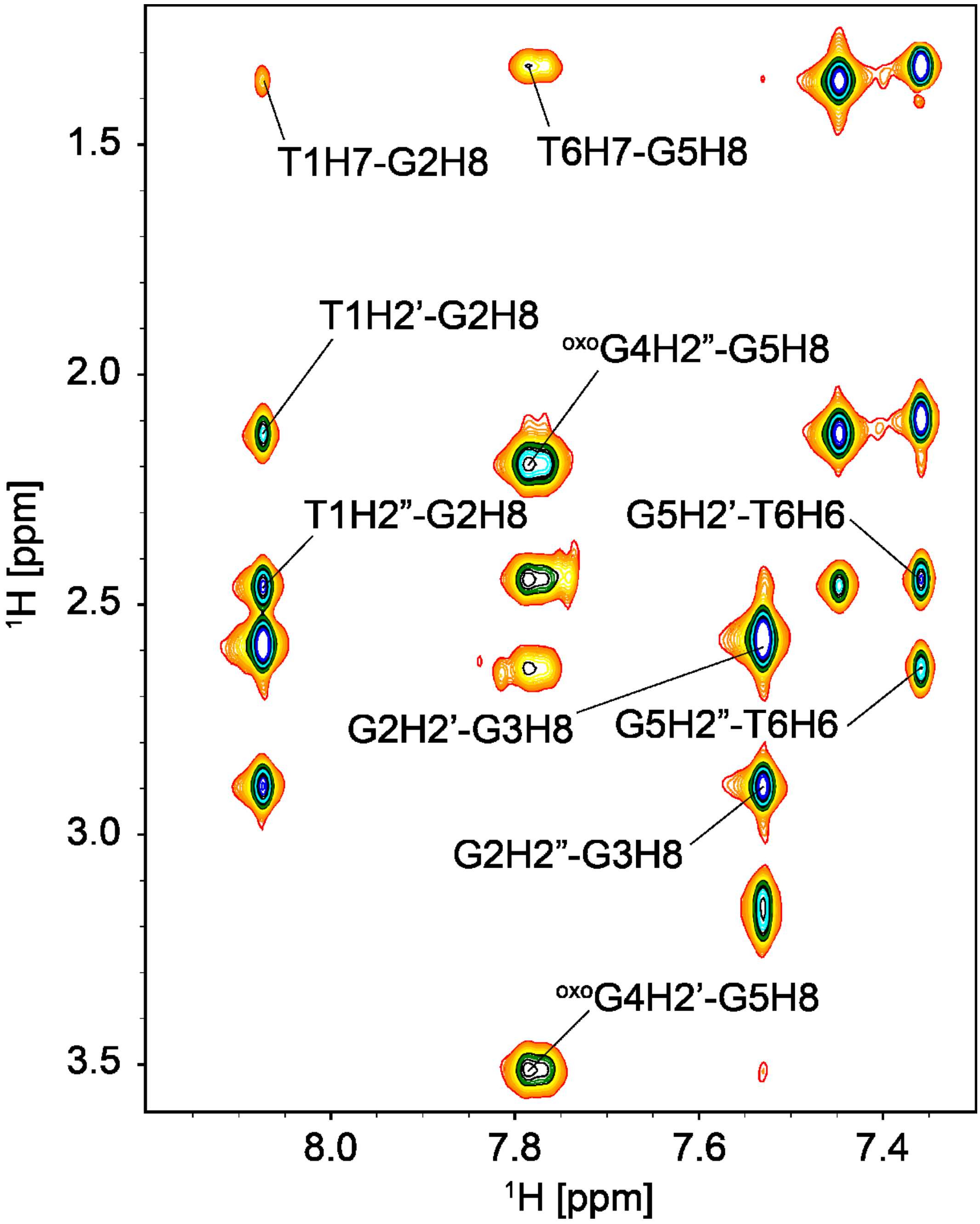

Figure S8: Aromatic-H7/H2'/H2'' region of a NOESY spectrum (τ_m = 250 ms, 25 °C) of ODN4 in 100 mM KCl, 10 mM KPi, pH 7 and 90%/10 % $^1H_2O/^2H_2O$. Concentration of oligonucleotide was 0.7 mM per strand. Intra-nucleotide peaks are not labeled.

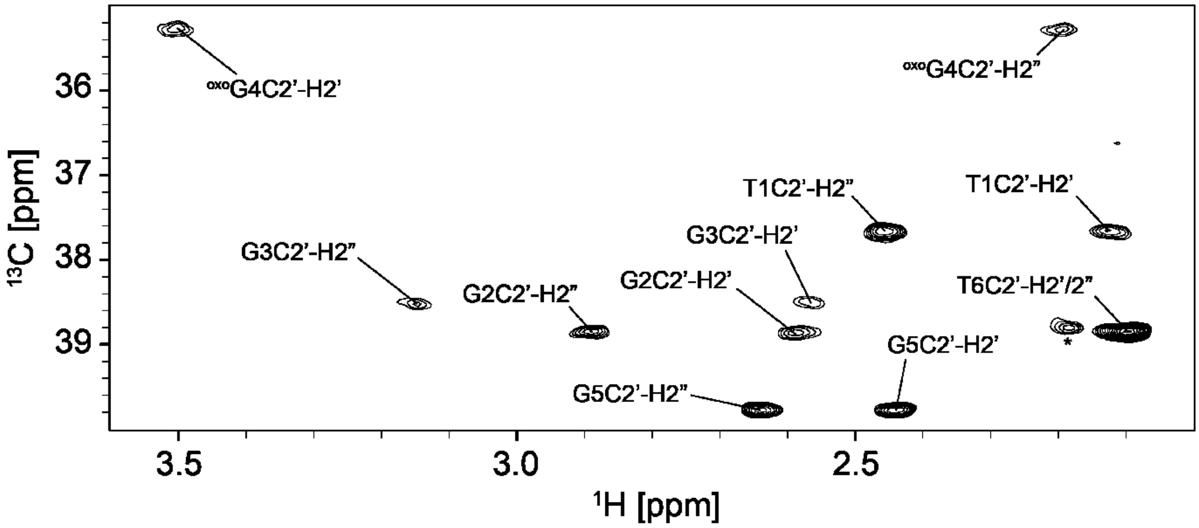

Figure S9. ^{13}C-edited HSQC spectrum of ODN4 in 100 mM KCl, 10 mM KPi, pH 7 and 90%/10 % $^{1}H_2O/^{2}H_2O$. Concentration of oligonucleotide was 0.7 mM per strand. Star-labeled peak is due to presence of a trace synthesis impurity

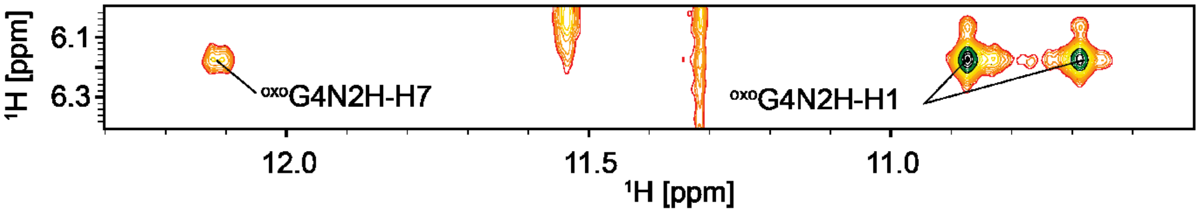

Figure S10. Imino-amino region of a NOESY spectrum (τ_m = 250 ms, 25 °C) of ODN4 at 25 °C in the presence of 100 mM KCl, 10 mM KPi, pH 7, 90 %/10% $^{1}H_2O/^{2}H_2O$. Concentration of oligonucleotide was 0.7 mM per strand.

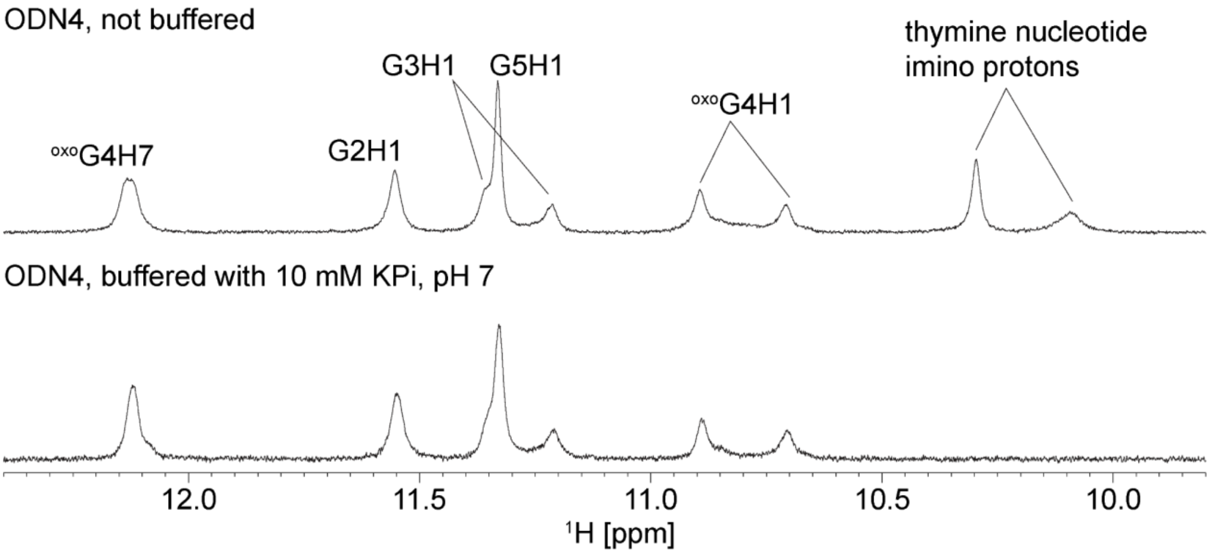

Figure S11. Imino region of ^{1}H 1D NMR spectrum of ODN4 at 25 °C. Spectrum was recorded on a 600 MHz spectrometer. Oligonucleotide in upper spectrum was dissolved in 100 mM KCl, 90%/10% $^{1}H_2O/^{2}H_2O$, with pH ≈ 5. Oligonucleotide in lower spectrum was dissolved in 100 mM KCl, 10 mM KPi, pH 7, 90%/10% $^{1}H_2O/^{2}H_2O$. Concentration of oligonucleotide was 0.7 mM per strand.

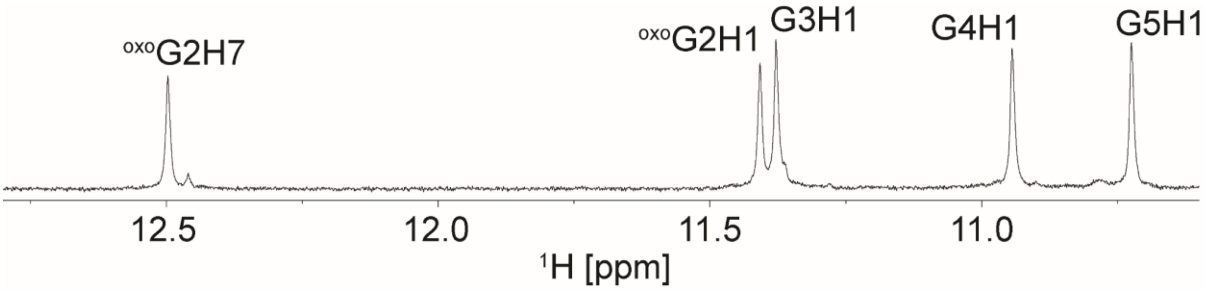

Figure S12. Imino region of ^{1}H 1D NMR spectrum of ODN2 at 5 °C. Spectrum was recorded on an 800 MHz spectrometer. Oligonucleotide was diluted in 100 mM KCl, 10 mM KPi, pH 7 with 90%/10% $^{1}H_2O$/$^{2}H_2O$. Concentration of oligonucleotide was 1.0 mM per strand.

Table S1. RMS pairwise difference between ten lowest energy simulated annealing structures of ODN2-5. Only the G-quadruplex core (nucleotides 2 to 5) was used in the calculation.

G-quadruplex	RMSD (Å)
ODN2	1.74
ODN3	1.40
ODN4	0.98
ODN5	1.81
